# Out-of-Hospital Surgical Airway Management: Does Scope of Practice Equal Actual Practice?

**DOI:** 10.5811/westjem.2016.3.28729

**Published:** 2016-05-05

**Authors:** Molly Furin, Melissa Kohn, Ryan Overberger, David Jaslow

**Affiliations:** * Albert Einstein Healthcare Network, Department of Emergency Medicine, Philadelphia, Pennsylvania; † Philadelphia University, Department of Emergency Medicine, Philadelphia, Pennsylvania

## Abstract

**Introduction:**

Pennsylvania, among other states, includes surgical airway management, or cricothyrotomy, within the paramedic scope of practice. However, there is scant literature that evaluates paramedic perception of clinical competency in cricothyrotomy. The goal of this project is to assess clinical exposure, education and self-perceived competency of ground paramedics in cricothyrotomy.

**Methods:**

Eighty-six paramedics employed by four ground emergency medical services agencies completed a 22-question written survey that assessed surgical airway attempts, training, skills verification, and perceptions about procedural competency. Descriptive statistics were used to evaluate responses.

**Results:**

Only 20% (17/86, 95% CI [11–28%]) of paramedics had attempted cricothyrotomy, most (13/17 or 76%, 95% CI [53–90%]) of whom had greater than 10 years experience. Most subjects (63/86 or 73%, 95% CI [64–82%]) did not reply that they are well-trained to perform cricothyrotomy and less than half (34/86 or 40%, 95% CI [30–50%]) felt they could correctly perform cricothyrotomy on their first attempt. Among subjects with five or more years of experience, 39/70 (56%, 95% CI [44–68%]) reported 0–1 hours per year of practical cricothyrotomy training within the last five years. Half of the subjects who were able to recall (40/80, 50% 95% CI [39–61%]) reported having proficiency verification for cricothyrotomy within the past five years.

**Conclusion:**

Paramedics surveyed indicated that cricothyrotomy is rarely performed, even among those with years of experience. Many paramedics felt that their training in this area is inadequate and did not feel confident to perform the procedure. Further study to determine whether to modify paramedic scope of practice and/or to develop improved educational and testing methods is warranted.

## INTRODUCTION

Emergency airway management has changed dramatically over the past few decades both in the emergency department (ED) and the prehospital setting. The widespread use of rapid sequence intubation, video laryngoscopy, and the development of multiple alternative airway devices have increased oral intubation success. The de-emphasis on endotracheal intubation in resuscitation algorithms has altered the setting in which airway management occurs. From the data in the National Emergency Medical Services Information System (NEMSIS), endotracheal intubation is typically performed once every 225 patient care incidents.^1^ Emergency medical services (EMS) protocols for the use of rapid sequence intubation and cricothyrotomy vary considerably across the country. The training, experience, and confidence levels of prehospital providers are perhaps equally as diverse. Cricothyrotomy is the definitive emergency technique to secure a patient’s airway when endotracheal intubation fails and the patient is unable to be ventilated by non-invasive methods. The incidence of surgical cricothyrotomy has declined in recent years and emergency providers may have less exposure to the procedure.^2^–^5^ This lack of clinical exposure and performance may affect the success rate and confidence level of providers in accomplishing the procedure.

While prior studies examine the prevalence and effectiveness of cricothyrotomy by helicopter EMS and in combat or austere environments, there is an inadequate amount of current literature to evaluate clinical competency in out-of-hospital surgical airway management by ground EMS crews.^6^–^9^ Since the procedure is so rare, it is difficult to use standard quality assurance methods to confirm competency. The purpose of this study was to assess clinical exposure, education, and self-perceived competency by ground paramedics in out-of-hospital surgical airway management.

## METHODS

This is a self-administered cross-sectional survey consisting of 22 questions regarding years of experience, surgical airway attempts, types of devices used, training in out-of-hospital surgical airway management, skills verification, and self-perceptions regarding procedural competence and comfort. This study was approved by the institutional review board at the authors’ institution.

The state of Pennsylvania includes out-of-hospital surgical airway management within the scope of practice of paramedics. This study includes four ground EMS agencies in four different counties within the state of Pennsylvania. The organizations include fire-based services and non-profit, municipal services. The number of annual emergency calls varies, with the largest agency reporting approximately 13,000 calls per year. The agencies serve areas that range from rural to suburban to urban communities.

Annual skills testing under the supervision of the medical director is required for paramedics to verify competency in necessary procedures and knowledge of state protocols. All actively practicing paramedics participate in the annual skills review. The out-of-hospital surgical airway management survey was self-administered during the course of the skills review for medical command reauthorization at the different agencies. The study included both full-time and part-time medics, as long as they were actively practicing in one of the participating 911 emergency response agencies. Medics who practice at more than one participating agency completed only one survey. The survey was self-administered during the skills review sessions and returned to the investigators at the end of each session.

We used Likert scales to design the survey. The outcome measures include number of cricothyrotomies performed and observed, the amount of training received in cricothyrotomies, and the providers’ comfort levels and confidence in performing the procedure. We compiled and then analyzed data using descriptive statistics, which are reported here (Microsoft Excel 2010).

## RESULTS

One hundred percent of ground paramedics actively practicing at one of the four agencies (86/86) responded to the survey. The cohort was heavily career staff with slightly more than half having more than 10 years of experience (Table 1) and most working more than 36 hours per week (Table 2). Only 20% of subjects had attempted out-of-hospital surgical airway management (17/86; 95% CI [11–28%]). Of these paramedics, 76% have greater than 10 years’ experience in the field (13/17; 95% CI [53–90%]). Of the 17 attempts at out-of-hospital surgical airway management five were by needle cricothyrotomy, 10 with commercial devices, and two by open cricothyrotomy.

**Figure 1:**
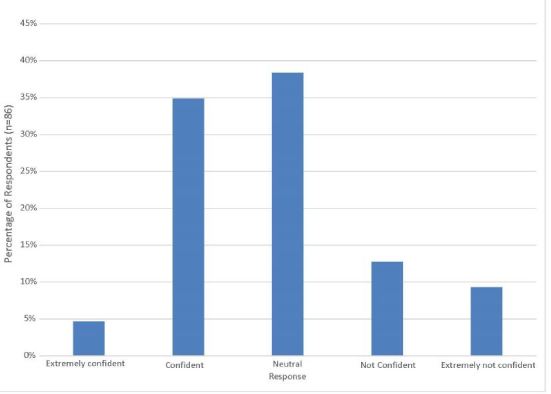
How confident do you feel that you could correctly perform a prehospital cricothyrotomy on the first attempt tomorrow?

Seventy-three percent of subjects indicated that they do not believe that they are trained well enough to perform out-of-hospital surgical airway management (63/86; 95% CI [64–82%]). Similarly, only 40% (34/86, 95% CI [30–50%]) were confident in their ability to perform cricothyrotomy correctly on the first attempt. Figure 1 displays provider responses when asked “which best describes how confident you would be that you could correctly perform a prehospital cricothyrotomy on the first attempt tomorrow?”

**Figure 2:**
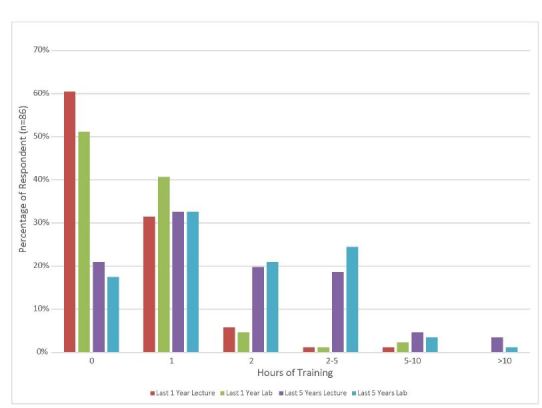
How much and what type of training have you had in prehospital cricothyrotomy in the past 1 year and the past 5 years?

Half of subjects with five or more years of experience had less than one hour of experience (39/70, 56%, 95% CI [44–68%]) per year of practical cricothyrotomy training within the last five years. Half of the subjects who were able to recall (40/80, 50% 95% CI [39–61%]) reported having proficiency verification for cricothyrotomy within the past five years. Figure 2 shows provider recall of the number of lecture and practical training hours in cricothyrotomy in the past one year and past five years.

## DISCUSSION

Principles of emergency airway management have evolved over the past several decades. Rapid sequence intubation has become commonplace in the ED, and the use of these techniques has been adopted in many prehospital systems. Numerous airway devices and alternative types of video and direct laryngoscopes have been developed and adopted by emergency physicians and prehospital providers. Most recently, the emphasis on obtaining what is considered a “definitive airway,” in most cases endotracheal intubation, has shifted to other aspects of resuscitative care. These changes have affected the setting in which paramedics practice and have likely altered the type of airway management they use.

Difficult airway algorithms often include needle or surgical cricothyrotomy when endotracheal intubation cannot be achieved by other means. Surgical cricothyrotomy has decreased in the nation’s EDs, which parallels the use of rapid sequence intubation, video laryngoscopy, and the use of supraglottic airways outside of the operating room.^5^ Similarly, as these techniques become more common in the prehospital setting, in particular neuromuscular blocking agents, this has also contributed to the diminishing frequency of cricothyrotomy.^3^ The results from this study demonstrate few attempts at cricothyrotomy even after years of practice. As the use of this procedure becomes less frequent, providers may become less competent to perform the procedure adequately. Establishment and maintenance of procedural competence require in-depth training and skills review. Naturally as prehospital providers become more skilled in difficult airway management, surgical airways become less frequent.^10^ In the increasingly rare cases where other methods of airway management fail, training of cricothyrotomy on mannequins may improve success rates and performance times.^11^–^13^ Competence, however, declines over time from initial training.^14^ In fact, it is recommended that refresher training be conducted at least every six months to maintain competence.^15^,^16^

Initial paramedic training may be brief yet includes multiple life-saving interventions and skills. Continuing education in many states, such as Pennsylvania, is variable and does not have required elements. While medical directors for EMS agencies must confirm paramedic skills annually, there is no uniform process by which to do so and much is left to the discretion of the particular medical director. The amount of training and continuing education that focuses on airway skills can be highly variable. Due to the brief amount of time paramedics may have to focus on continuing education and the maintenance of skills, it is crucial that the most valuable interventions be emphasized and these procedural skills be assessed regularly. If paramedics are not performing this skill in the field, and are not receiving recurrent education and training, their ability to successfully complete the procedure is probably reduced. While this study did not directly assess paramedics’ ability to successfully complete a surgical cricothyrotomy, it did evaluate their comfort level and self-confidence to perform the procedure. The majority of the paramedics did not feel trained well enough to perform an out-of-hospital cricothyrotomy, and most did not believe that they could perform it correctly on the first attempt.

A meta-analysis of 56 studies by Hubble et al reported needle cricothyrotomy success rates of 65.8% and surgical cricothyrotomy success rates of 90.5%, with the physician success rate slightly higher at 97.1%.^17^ Paramedic training and skills education were variable in many of these studies though, and results may not be able to be generalized to the broader group of ground paramedics. Some studies report six hours of initial didactic sessions with added airway labs, and others require refresher procedure labs annually or every two years.^15^,^18^,^19^ The results from this survey show that this population, including four services from diverse areas of Pennsylvania, did not receive that amount of education or skills re-verification. This broad sampling may be more similar to other groups of ground paramedics. In this setting, success rates of cricothyrotomies may be significantly less than those reported in prior studies. While variability exists in the performance of surgical airway management, it is clear from existing literature that the procedure is typically reserved for the most critical patients. Several studies show extremely poor survival rates, and even fewer patients who survive with good neurologic outcomes.^15^,^18^,^20^,^21^ The amount of time and training to establish and maintain procedural competency among ground paramedics must be weighed against the perceived potential for positive patient outcomes.

## LIMITATIONS

Certain limitations exist that limit the utility of this dataset. Cricothyrotomy is rarely performed, and therefore it is difficult to study prospectively or to analyze outcomes. Survey methodology was deemed the most appropriate study design in order to assess paramedics’ previous training, confidence, and experience in performing the skill. Currently, there is no validated survey available on this specific topic. Given the staff turnover rate at many EMS agencies, reproducing the study with the same subjects would be nearly impossible to perform. It was understood that in performing this type of study that reliability would be a limitation.

This study relied on paramedics’ recollections of previous patient encounters and is therefore subject to recall bias. Success of the procedure was subjectively determined by the paramedics and is understood that clear parameters for success could not be established. Paramedics are likely to recall attempting to perform a cricothyrotomy due to the unique and stressful circumstances. They may have incorrect recollections about the amount of training on cricothyrotomies they received. However, it was deemed more important to assess paramedics’ subjective recollections about their education and training rather than a medical director’s perception as to whether or not the skill had been taught.

This survey attempted to characterize a paramedic’s own perceptions regarding an infrequently used skill in airway management. Medics often work with several different squads and may have received additional training at an outside agency, which would have affected their confidence levels; however, this training would also be reported in the survey. To ensure maximal response rate and limit sampling bias, the survey was only distributed during in-person training sessions, which resulted in a smaller number of subjects. This was mitigated by including diverse agencies in four counties, but as a result the study may not be representative of agencies across the state of Pennsylvania or in other states. Additional investigations with a larger study population in other EMS systems may be warranted.

## CONCLUSION

Cricothyrotomy is an infrequently performed intervention and patient outcomes are typically poor. Prehospital providers have limited time and access to continuing education and training sessions and must be relied upon to deliver medical care that improves patient outcomes. The responses in this study demonstrate that paramedics may benefit from additional training and recurrent education and skills verification.

## Figures and Tables

**Table 1:** Respondent experience of paramedics in out-of-hospital surgical airway management study (years).

Experience (years)	Paramedics (number)
0 to 5	20
6 to 10	18
11 to 15	19
16 to 20	11
21 to 25	6
>26	10

**Table 2:** Respondent experience (hours).

On-duty time (hrs/week)	Paramedics (number)
0 to 20	8
21 to 35	3
36 to 50	41
>51	32
